# Dissecting the sub-structure of the intraflagellar transport complex B

**DOI:** 10.1186/2046-2530-1-S1-P51

**Published:** 2012-11-16

**Authors:** Y Texier, J van Reeuwijk, DA Mans, R Roepman, K Boldt, M Ueffing

**Affiliations:** 1 Medical Proteome Center, Center of Ophthalmology, University of Tuebingen, Germany; 2 Nijmegen Centre for Molecular Life Sciences, Radboud University Nijmegen Medical Centre, the Netherlands; 3 Helmholtz Zentrum Muenchen, German Research Center for Environmental Health (GmbH), Research Unit Protein Science, Neuherberg, Germany

## 

The intraflagellar transport (IFT) machinery is composed of mainly of two major components, IFT complex A and B as well as of motor proteins (kinesins and dyneins). The aim of this study was to identify comprehensively the composition of the IFT complex B as well as the structure of its functional sub-modules. We applied Strep/FLAG-tandem affinity purification (SF-TAP) and yeast-two-hybrid to identify protein complexes and protein-protein interactions within IFT complex B. By combining these methods with the biochemical separation and destabilization of the protein complex B into sub-complexes and mass spectrometric analysis, we further determined its structure. As a first step, we comprehensively identified the composition of the IFT complex B by SF-TAP using several IFT complex B proteins as baits. The sub-complex analysis by SDS-destabilization and sucrose-density gradient centrifugation revealed that this complex is composed of at least two stable sub-complexes. The analysis further revealed that these two sub-complexes are likely to be connected by two IFT complex B proteins that either are present in both sub-complexes, or are excluded from both but act as a linker. These data suggest, that the IFT complex B is not, as previously described, acting as a single stable complex with proteins associated to the core structure. The biochemical analysis of the sub-complex structure shows, that there are two sub-modules that are closely linked. It remains unclear, if these sub-complexes exert one function as a tandem, or if they can act as separated modules within cilia or possibly within other microtubular structures.

